# Mediating effect of suicidal ideation in the association between child abuse and premenstrual syndrome among female adults

**DOI:** 10.1186/s12905-024-02949-9

**Published:** 2024-02-07

**Authors:** Maya Kfoury, Diana Malaeb, Perla Moubarak, Fouad Sakr, Mariam Dabbous, Souheil Hallit, Feten Fekih-Romdhane, Sahar Obeid

**Affiliations:** 1 School of Arts and Sciences, Holy Spirit University of Kaslik, P.O. Box 446, Jounieh, Lebanon; 2 College of Pharmacy, Gulf Medical University, Ajman, United Arab Emirates; 3 School of Medicine and Medical Sciences, Holy Spirit University of Kaslik, P.O. Box 446, Jounieh, Lebanon; 4 School of Pharmacy, Lebanese International University, Beirut, Lebanon; 5 École Doctorale Sciences de la Vie et de la Santé, Université Paris-Est Créteil, Institut Mondor de Recherche Biomédicale, Créteil, France; 6 Applied Science Research Center, Applied Science Private University, Amman, Jordan; 7 The Tunisian Center of Early Intervention in Psychosis, Department of psychiatry “Ibn Omrane”, Razi hospital, Manouba, 2010 Tunisia; 8 Faculty of Medicine of Tunis, Tunis El Manar University, Tunis, Tunisia; 9 Social and Education Sciences Department, School of Arts and Sciences, Lebanese American University, Jbeil, Lebanon

**Keywords:** Child abuse, Premenstrual symptoms, Suicidal ideation, Trauma-informed care, Mental health

## Abstract

**Introduction:**

Premenstrual symptoms encompass a range of physical, emotional, and behavioral changes that cyclically occur before menstruation. Childhood abuse has been associated with subsequent mental health challenges, yet its relationship with exacerbating premenstrual symptoms remains an understudied area. Furthermore, suicidal ideation often emerges from traumatic backgrounds such as child abuse, creating another layer of complexity. Given the rising suicide rates in Lebanon, and the concurrent increase in reported child abuse cases, this research focuses on the role of suicidal ideation as a mediator between child abuse and premenstrual syndrome.

**Methods:**

This cross-sectional study involved 915 female university students in Lebanon. Participants completed an online questionnaire encompassing demographic details, health lifestyle, the Premenstrual Symptoms Screening Tool (PSST), Columbia-Suicide Severity Rating Scale (C-SSRS), and Child Abuse Self Report Scale (CASRS-12). The mediation analysis was conducted using PROCESS MACRO v3.4 model 4; three pathways derived from this analysis: pathway A from the independent variable to the mediator, pathway B from the mediator to the dependent variable, Pathway C indicating the direct effect from the independent to the dependent variable.

**Results:**

The results of the mediation analysis showed that suicidal ideation mediated the association between all types of child abuse and the presence of PMS. Higher psychological (Beta = 0.21; *p* < 0.001), neglect (Beta = 0.02; *p* = 0.017), physical (Beta = 0.19; *p* < 0.001) and sexual (Beta = 0.20, *p* < 0.001) child abuse were significantly associated with higher suicidal ideation, which was significantly associated with the presence of PMS (Beta = 0.38, *p* = 0.001; Beta = 0.57, *p* < 0.001; Beta = 0.45, *p* < 0.001; and Beta = 0.50, *p* < 0.001) respectively. Finally, higher psychological (Beta = 0.17, *p* < 0.001), physical (Beta = 0.11, *p* = 0.024), but not sexual (Beta = 0.07, *p* = 0.198) child abuse was directly and significantly associated with the presence of PMS, whereas higher neglect (Beta = -0.06, *p* = 0.007) was significantly associated lower odds of having PMS.

**Conclusion:**

This study highlights the mediating role of suicidal ideation in the complex association between different types of childhood abuse and premenstrual symptoms. The findings emphasize the need for trauma-informed care and tailored interventions to address the diverse impact of these factors. Recognizing the intricate relationships between child abuse, suicidal ideation, and PMS can aid healthcare providers in comprehensively addressing young women’s mental and reproductive well-being. Trauma-informed care, tailored interventions and awareness of potential connections between childhood maltreatment are essential in managing these complex challenges.

## Introduction

Premenstrual syndrome (PMS) refers to a cyclic and recurrent cluster of physical, emotional, and/or behavioral symptoms that occur prior to menstruation during the luteal phase, and tend to diminish within a few days after the onset of menses [1]. These symptoms, varying among women and cycles, include fatigue, mood swings, irritability, cravings, headaches, and body aches [2]. . The precise etiology and pathogenesis have yet to be fully determined, but several theories, including hormonal fluctuations (progesterone, estrogen), and neurotransmitter imbalances (serotonin, GABA, endorphins) have been proposed to explain this condition. Genetic predisposition, and environmental lifestyle factors may also contribute to the exacerbation of these symptoms [3].Premenstrual symptoms are intertwined with various aspects of physical and mental wellbeing [4]. These symptoms often normalized, can significantly impact the quality of life and productivity of young women, their academic performance, their family relationships, and their social engagement [5, 6]. Moreover, research has indicated correlations between factors such as sleep disturbances, depressive thoughts, mood changes, and the severity of premenstrual symptoms, highlighting their impact on overall wellbeing [7].

Adverse childhood experiences and trauma have been identified as factors related to the manifestation of premenstrual symptoms [4, 8]. According to The World Health Organization (WHO) [9], child maltreatment is a worldwide public health concern encompassing physical ill-treatment, emotional ill-treatment, sexual abuse, physical and / or emotional neglect, and exploitation. It inflicts actual or potential harm on a child’s health, survival, development and dignity [10]. Research underscores the connection between childhood maltreatment and premenstrual symptoms. Notably, childhood emotional and physical abuse significantly elevate the risk of experiencing PMS. Additionally, although to differing extents, neglect and sexual abuse also demonstrate associations with the occurrence of PMS [11]. Furthermore, previous findings [12] have indicated that the number and severity of premenstrual symptoms increased with greater exposure to childhood trauma. This relationship was found to be completely mediated by difficulties in regulating emotions [12]. In contrast, other studies have explored a different dimension of this connection; they suggested that the influence of childhood maltreatment, particularly neglect, on premenstrual mental symptoms is mediated by affective temperaments [13]. Within the framework of the relationship between child maltreatment and premenstrual disturbances, it remains intriguing to consider an additional factor, i.e. the presence of suicidal ideation.

Suicidal ideation is an umbrella term used to describe a range of contemplations, wishes, and preoccupation with death and suicide [14]. The premenstrual phase has been identified as a period of heightened risk for suicide [15]. Women diagnosed with premenstrual disorders face elevated risks of experiencing suicidal ideation, suicide attempts, and formulating suicide plans [15–17]. [18, 19]. . Studies indicate a correlation between suicide behavior and specific premenstrual symptoms. Suicide attempts were notably linked to irregular periods and increased appetite. For instance, suicidal ideation showed connections with irregular periods, low back pain, increased appetite, guilt, and aggressiveness [20]. Moreover, approximately half of females attempting suicide have reported experiencing PMS. Previous research has notably linked childhood abuse to the development of suicidal thoughts, often mediated by underlying psychological factors [8, 21]. Research from 79 studies revealed strong links between childhood maltreatment (including emotional, physical, sexual abuse, neglect, and combined abuse) and heightened suicide risk in young individuals. These experiences raised the odds of suicidal thoughts, with sexual abuse leading to a fourfold increase in planning [22]. Another meta-analysis of 68 studies consistently associated childhood maltreatment with elevated suicide risk in adulthood. Notably, repetitive and combined abuse showed a fourfold increase in planning [23]. Emotional abuse emerged as a significant factor in the pathway to suicidal tendencies. Concerning the connections between the physical, psychological, sexual abuse and women’s experience of premenstrual symptoms, the following explanations can be proposed. Childhood traumatic experiences can lead to a cascade of physiological and psychological responses that may, in turn, indirectly influence the severity and perception of PMS symptoms. For one, the chronic stress and trauma associated with abuse can disrupt the body’s hormonal balance, potentially exacerbating hormonal fluctuations responsible for PMS symptoms [24]. Survivors of abuse often develop heightened sensitivity to physical emotional changes, which may be lead to increased awareness and reporting of PMS symptoms [25]. Furthermore, coping mechanisms developed in response to abuse, such as unhealthy eating habits or substance abuse, can indirectly contribute to hormonal imbalances or mood swings commonly associated with PMS [26, 27].

As culture has been shown to influence the premenstrual experience [28, 29], cultural norms around the menstrual cycle may influence Lebanese’s women’s experiences of premenstrual symptoms. Discussing negative aspects of PMS is often avoided due to cultural taboos, potentially leading to the activation of emotional schemas of deprivation, isolation, defectiveness, and negativity. This context is essential when considering the psychological well-being of Lebanese women facing premenstrual issues [30]. On the other hand, the alarming rise in suicide rates in Lebanon [31, 32], coupled with a simultaneous increase in reported cases of child abuse [33, 34], underscore an urgent need to uncover the intricate connections between these critical issues. Amid a growing awareness of the harmful impact of child maltreatment on mental health, an unexplored area lies in understanding its potential impact on both premenstrual disturbances and the emergence of suicidal ideation. This holds the potential to provide crucial insights into effective intervention strategies, treatment plans, and policy development, addressing the complex challenges experienced by young women in Lebanon. This research aims to explore the pathways between childhood abuse, suicidal ideation and PMS among young female adults. It is hypothesized that suicidal ideation will act as a mediator in the relationship between each type of childhood abuse (i.e., Psychological abuse, neglect, physical abuse, sexual abuse) and PMS.

## Methods

### Study design and participants

Utilizing a snowball sampling technique, a survey was generated using Google Forms and distributed through messaging platforms and social media networks, including WhatsApp, Instagram, and Messenger. The survey enlisted 915 respondents between January and May 2023. The criteria for participation were as follows: (1) being a resident and citizen of Lebanon, (2) age 18 or above, and (3) willingness to partake in the research. Those who declined to fill out the questionnaire were excluded. Following the provision of digital informed consent, participants were instructed to complete the aforementioned assessment tools, which were presented in a pre-determined sequence to mitigate order-related biases. The survey was conducted anonymously, and participants volunteered to participate without compensation, typically spending around 20 min on the survey.

### Minimal sample size calculation

According to Fritz and MacKinnon [35], a minimal sample of 412 was deemed necessary based on this formula: $n=\frac{L}{f2}+k+1$, where f=0.14 for small effect size, L=7.85 for an α error of 5% and power β = 80%, and k=10 variables (marital status, education level, cigarette smoking, child abuse, regular menses, oral contraceptives use, age, body mass index, financial burden and physical activity) to be entered in the model.

### Questionnaire

The survey tool (Appendix 1 at the end of the manuscript) utilized for data collection was an organized online questionnaire designed through Google Form in Arabic, Lebanon’s native language. Taking approximately 20 min to complete, the questionnaire was structured into various segments:

- Sociodemographic questions (marital status, education and age), financial burden (self-reported on a scale from 1 to 10, with 10 indicating heaviest burden), Body Mass Index (calculated according to the World Health Organization formula [36]. Physical activity index (calculated by multiplying the duration (1 = less than 10 min to 4 = 30 min or more) by the strength (1 = light activity to 5 = Heavy breathing and constant sweating) by the frequency (1 = less than once a month to 5 = daily or almost daily) of the physical activity [37]; scores varied between 3 and 100, with higher scores reflecting higher physical activity).
- Questions related to health and current lifestyle (cigarette smoking, regular menses, oral contraceptives).
*-The Premenstrual Symptoms Screening Tool (PSST)*, which is a retrospective questionnaire, helps distinguish women who experience severe PMS and PMDD [38]. Women use a 4-point Likert scale to measure the magnitude of their symptoms over the previous year (1 = none, 2 = mild, 3 = moderate, 4 = severe). The scale is divided into two parts, the first of which contains 12 PMS symptoms. E.g. *“during the week or two before your period, do you experience mood swings, irritability, or sudden mood changes?”* If the respondent selects “yes” for at least one symptom (i.e. 2–4 points), she must complete section B, which consists of five questions about menstruation-related disturbance of activities, behaviors or relationships. The total score is calculated by summing the answers to the 17 items (range between 17 and 68). The Arabic version has been previously used [39]. In this study, the Cronbach’s alpha was 0.94.
- *Columbia-Suicide Severity Rating Scale (C-SSRS).* This 6-Item instrument is used to evaluate suicidal ideation and behavior. E.g., *“have you ever wished you were dead or wished you could go to sleep and not wake up?”* Questions 1 to 5 evaluate suicidal behavior over the past month, while question 6 evaluates it over the respondent’s lifetime and past 3 months. Answering “yes” to any of the 6 questions indicates the presence of suicidal ideation. This scale is validated in Lebanese adults and adolescents [32, 40]. In this study, the Cronbach’s alpha was 0.72.
- *Child Abuse Self Report Scale (CASRS-12)* is a brief Arabic version of the Child Abuse Self Report Scale. It has been validated in Lebanon and is divided into 4 categories: Psychological (3 items*) E.g. “as a child, did someone often belittle you, insult you, or make you feel worthless?”*, Neglect (3 items) E.g. *“during your childhood, did you frequently go without proper food, clothing, or shelter?”*, physical abuse (3 items) E.g. *“during your childhood, did someone hurt you in a way that left bruises, marks, or injuries?”*, and sexual abuse (3 items) E.g. *“were you touched inappropriately by an adult when you were a child?”.* The responses are scored from 0 = Never to 3 = Always. In all subscales, higher scores point out more abuse [41]. The Cronbach’s alpha values were as follows: psychological abuse (0.86), neglect (0.85), physical abuse (0.82) and sexual abuse (0.89).

### Statistical analysis

The SPSS software v.25 was used for the statistical analysis. Cronbach’s alpha values were calculated for reliability purposes. Descriptive analysis were done as frequency/percentage for categorical variables and mean/standard deviation for continuous variables. The Chi-square test was used to compare two categorical variables, whereas the Student *t* test was used to compare two means. The mediation analysis was conducted using PROCESS MACRO (an SPSS add-on) v3.4 model 4; three pathways derived from this analysis: pathway A from the independent variable to the mediator, pathway B from the mediator to the dependent variable, Pathway C indicating the direct effect from the independent to the dependent variable. Results were adjusted over all variables that showed a *p* < 0.25 in the bivariate analysis. We considered the mediation analysis to be significant if the Boot Confidence Interval did not pass by zero. *P* < 0.05 was deemed statistically significant.

## Results

Nine hundred fifteen women completed the survey, with a mean age of 27.09 ± 9.28 years (min = 18; max = 50). The majority of the participants were single (69.0%), have a university level of education (82.4%), had PMS (59.3%), had regular menses (76.4%) and did not use oral contraceptives (91.1%). All characteristics of the sample are summarized in Table 1.

**Table 1:** Sociodemographic and other characteristics of the sample (*n* = 915)

	n (%)
Marital status	
Single	631 (69.0%)
Married	284 (31.0%)
Education	
Secondary or less	161 (17.6%)
University	754 (82.4%)
Cigarette smoking	
No	719 (78.6%)
Yes	196 (21.4%)
Presence of PMS	
No	372 (40.7%)
Yes	543 (59.3%)
Regular menses	
No	216 (23.6%)
Yes	699 (76.4%)
Oral contraceptives	
No	834 (91.1%)
Yes	81 (8.9%)
	**Mean ± SD**
Age (years)	27.09 ± 9.28
Body Mass Index (kg/m^2^)	23.57 ± 4.42
Financial burden	5.22 ± 2.41
Physical activity	22.61 ± 18.29
Psychological abuse*	1.02 ± 1.88
Neglect*	4.27 ± 2.97
Physical abuse*	0.81 ± 1.70
Sexual abuse*	0.58 ± 1.55
Suicidal ideation**	0.37 ± 0.88

*calculated using the Child Abuse Self Report Scale (CASRS-12). **calculated using the Columbia-Suicide Severity Rating Scale (C-SSRS).

### Bivariate analysis

A significantly higher percentage of participants who are single and have a university level of education was found in participants with PMS. Moreover, a significantly higher mean suicidal ideation, psychological, physical and sexual abuse, as well as a lower mean age and neglect were significantly found in participants with PMS (Table 2).

**Table 2:** Bivariate analysis of factors associated with presence vs. absence of premenstrual symptoms (PMS).

	Absence of PMS	Presence of PMS	p	Statistical test	Unadjusted odds ratio [95% CI]
Marital status			**< 0.001**	Chi-square	
Single	231 (36.6%)	400 (63.4%)			0.59 [0.44; 0.78]
Married	141 (49.6%)	143 (50.4%)			
Education			**0.003**	Chi-square	
Secondary or less	82 (50.9%)	79 (49.1%)			1.66 [1.18; 2.34]
University	290 (38.5%)	464 (61.5%)			
Cigarette smoking			0.479	Chi-square	
No	288 (40.1%)	431 (59.9%)			0.89 0.65; 1.23
Yes	84 (42.9%)	112 (57.1%)			
Regular menses			0.215	Chi-square	
No	80 (37.0%)	136 (63.0%)			0.82 0.60; 1.12
Yes	292 (41.8%)	407 (58.2%)			
Oral contraceptives			0.467	Chi-square	
No	336 (40.3%)	498 (59.7%)			0.84 0.53; 1.34
Yes	36 (44.4%)	45 (55.6%)			
Age	28.92 ± 10.40	25.84 ± 8.20	**< 0.001**	Student t test	0.97 [0.95; 0.98]
Body Mass Index	23.49 ± 4.10	23.62 ± 4.63	0.656	Student t test	1.01 [0.98; 1.04]
Financial burden	5.04 ± 2.37	5.35 ± 2.43	0.056	Student t test	1.06 [1.00; 1.12]
Physical activity	22.53 ± 17.96	22.67 ± 18.52	0.911	Student t test	1.00 [0.99; 1.01]
Psychological abuse	0.60 ± 1.37	1.31 ± 2.12	**< 0.001**	Student t test	1.27 [1.16; 1.39]
Neglect	4.56 ± 3.17	4.07 ± 2.82	**0.016**	Student t test	0.95 [0.91; 0.99]
Physical abuse	0.56 ± 1.36	0.99 ± 1.89	**< 0.001**	Student t test	1.18 [1.08; 1.29]
Sexual abuse	0.42 ± 1.32	0.70 ± 1.67	**0.005**	Student t test	1.14 [1.03; 1.25]
Suicidal ideation	0.18 ± 0.60	0.50 ± 1.01	**< 0.001**	Student t test	1.74 [1.40; 2.16]

Numbers in bold indicate significant *p* values.

### Mediation analysis

**Fig. 1:**
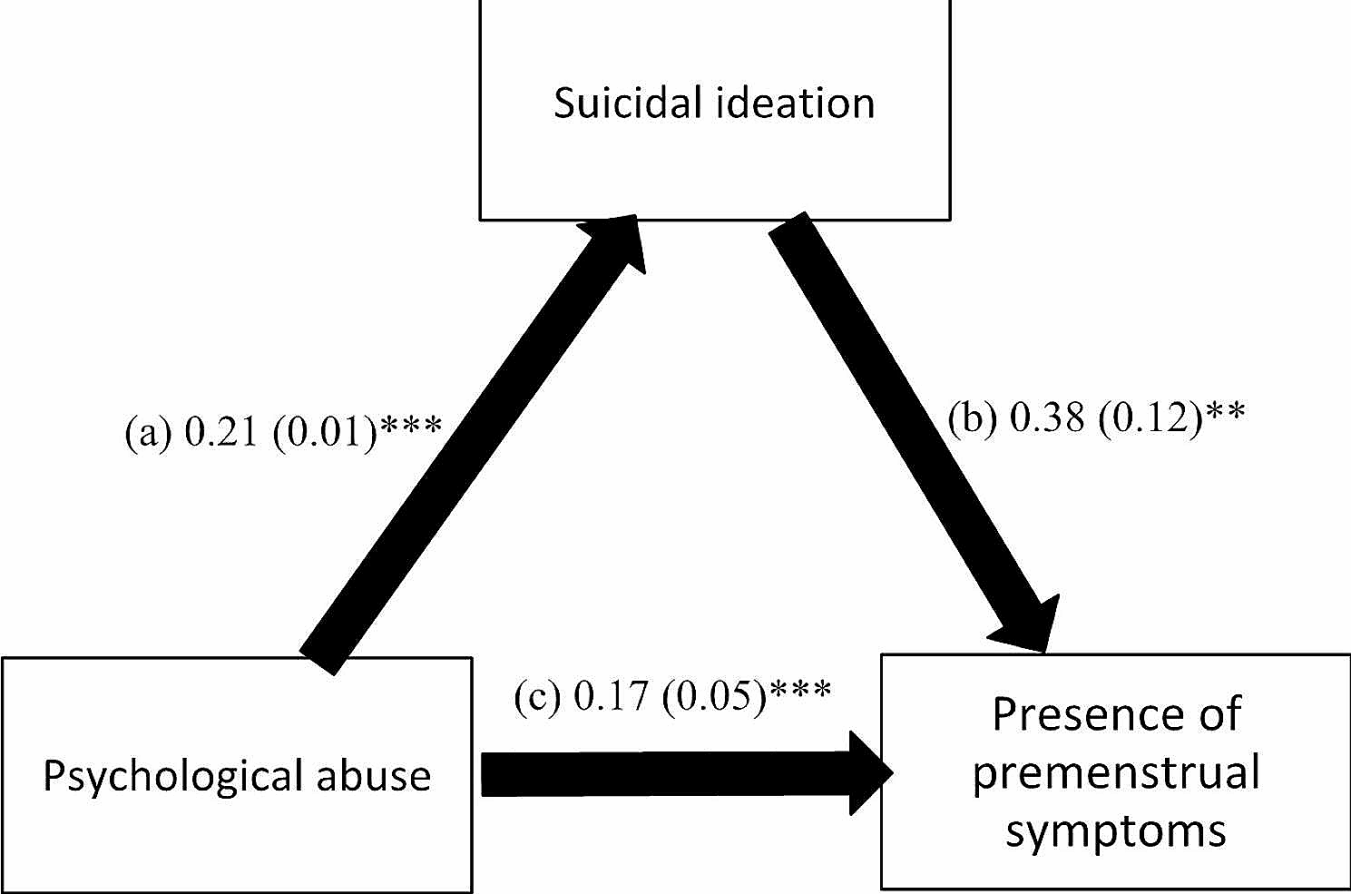
(a) Relation between psychological abuse and suicidal ideation (R^2^ = 0.230); (b) Relation between suicidal ideation and the presence of premenstrual symptoms (R^2^ = 0.106); (c) Direct effect of psychological abuse and the presence of premenstrual symptoms. Numbers are displayed as regression coefficients (standard error). ** *p* < 0.01; ***p < 0.001

**Fig. 2:**
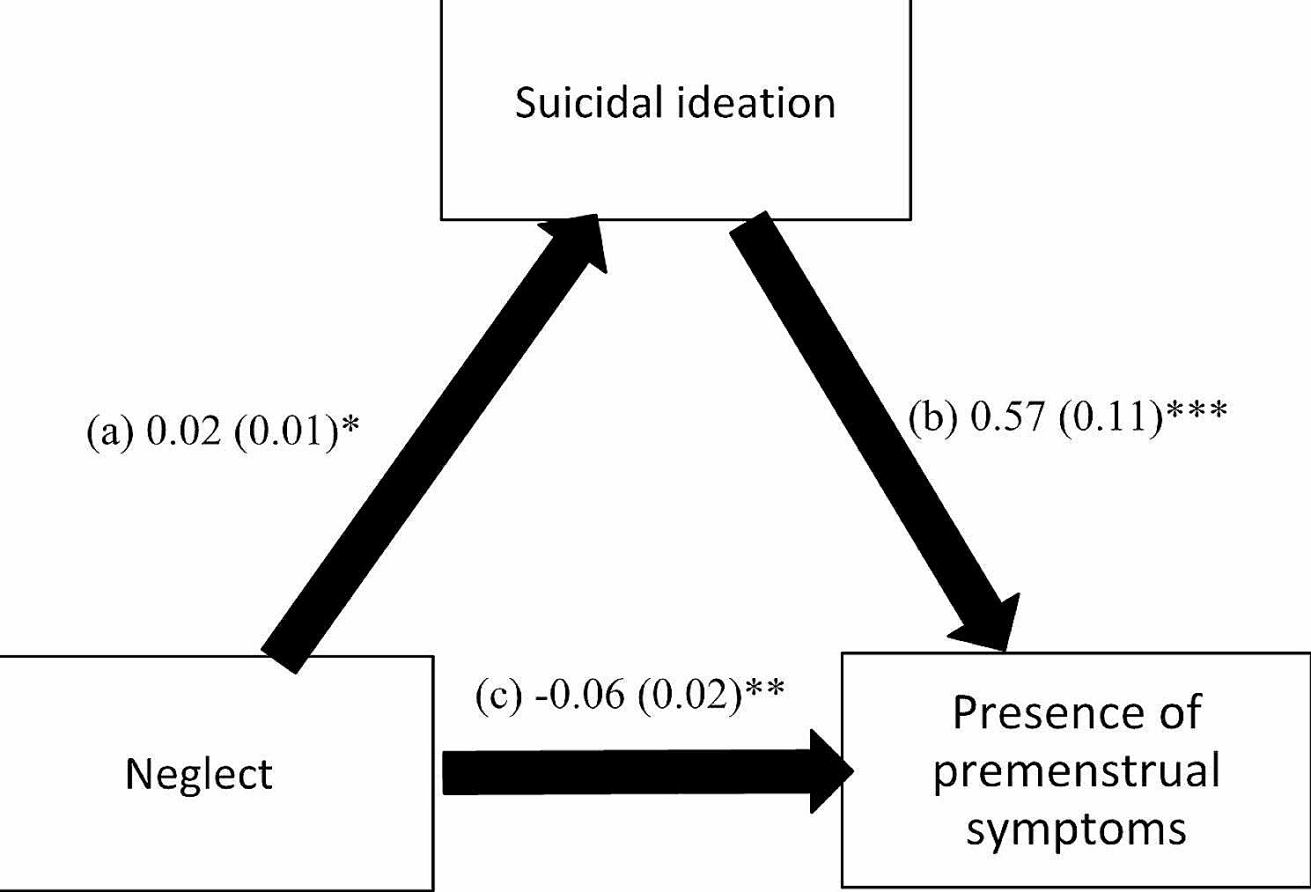
(a) Relation between neglect and suicidal ideation (R^2^ = 0.038); (b) Relation between suicidal ideation and the presence of premenstrual symptoms (R^2^ = 0.099); (c) Direct effect of neglect and the presence of premenstrual symptoms. Numbers are displayed as regression coefficients (standard error). * *p* < 0.05; ** *p* < 0.01; ***p < 0.001

**Fig. 3:**
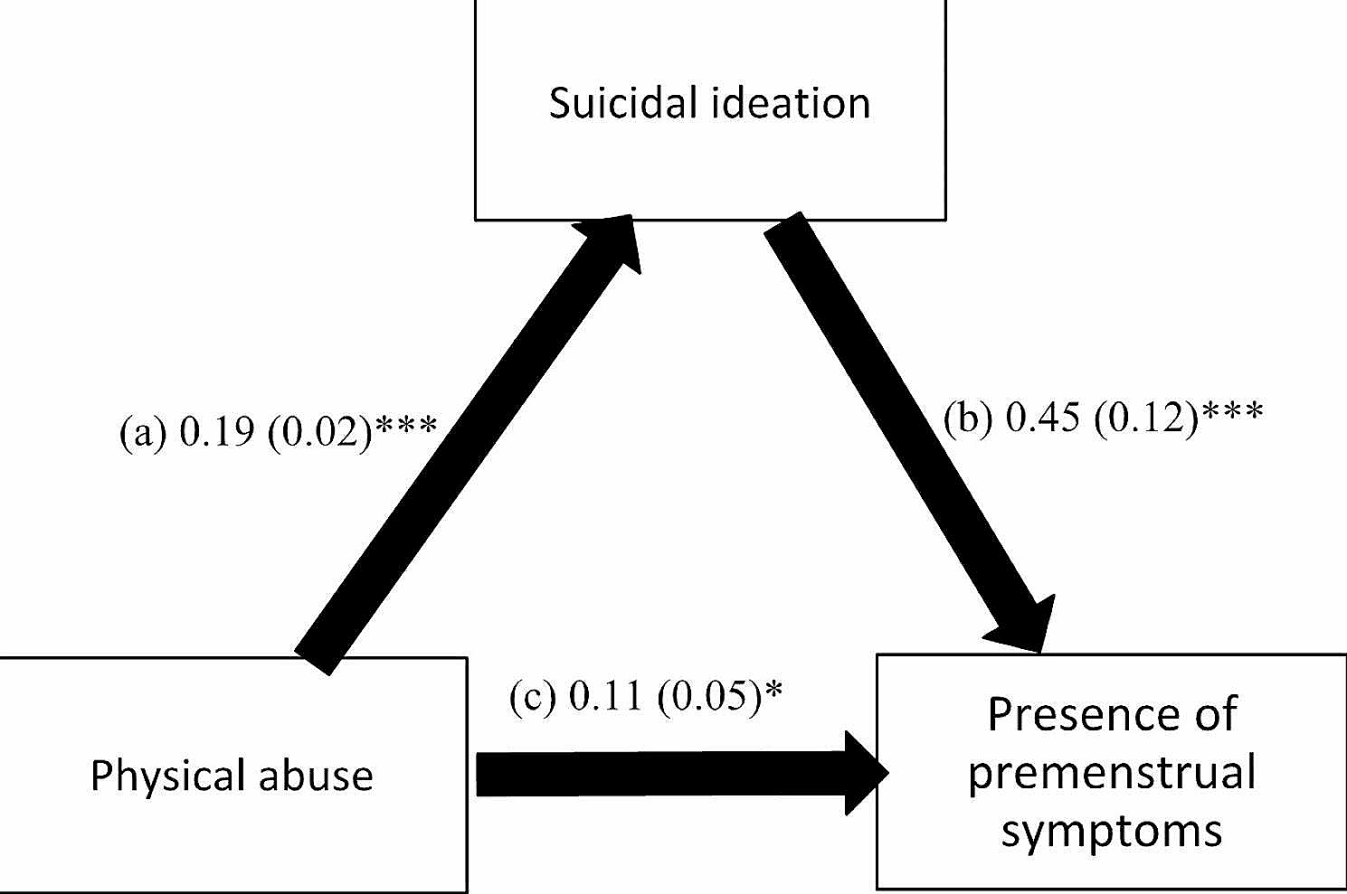
(a) Relation between physical abuse and suicidal ideation (R^2^ = 0.168); (b) Relation between suicidal ideation and the presence of premenstrual symptoms (R^2^ = 0.096); (c) Direct effect of physical abuse and the presence of premenstrual symptoms. Numbers are displayed as regression coefficients (standard error). * *p* < 0.05; ***p < 0.001

**Fig. 4:**
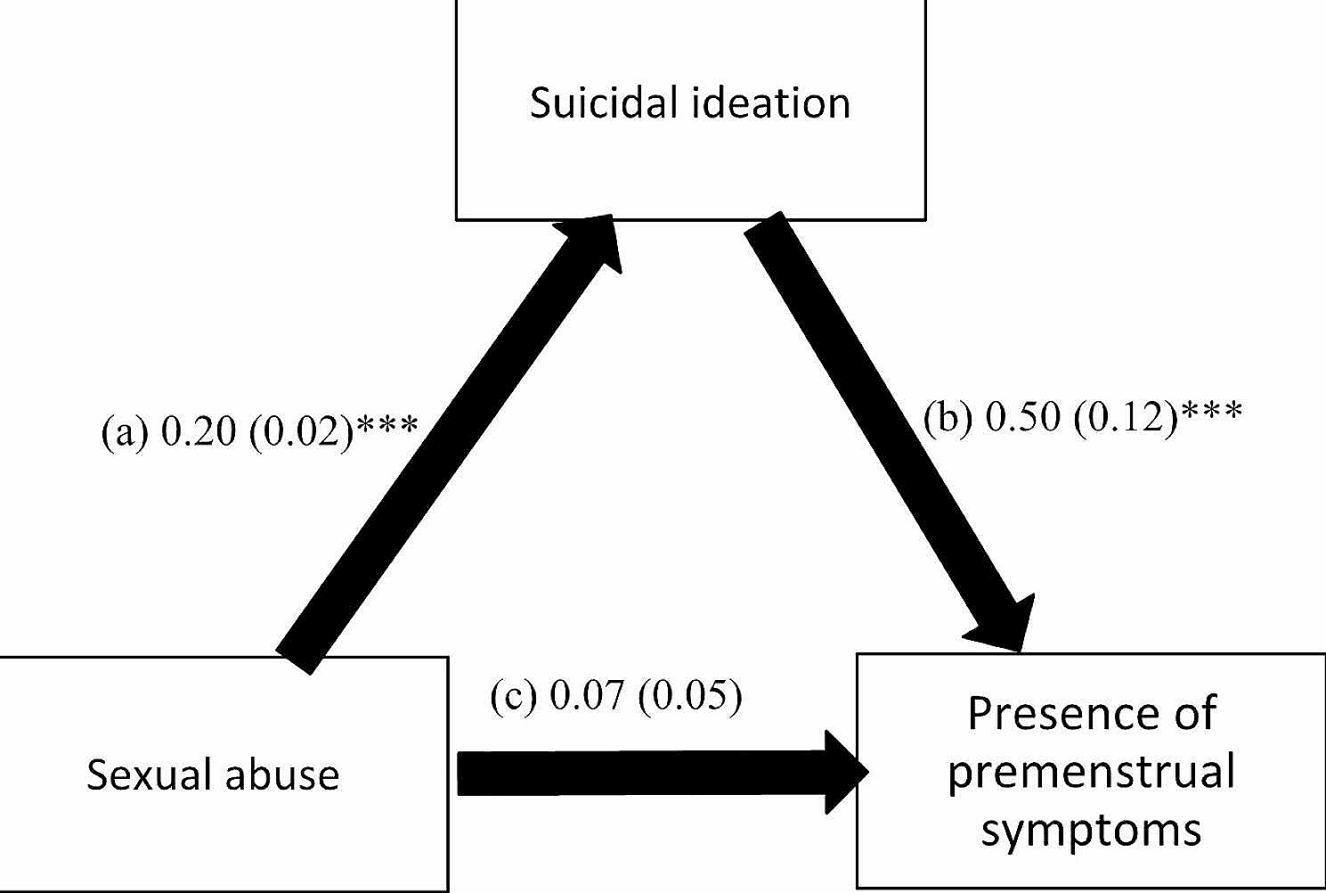
(a) Relation between sexual abuse and suicidal ideation (R^2^ = 0.149); (b) Relation between suicidal ideation and the presence of premenstrual symptoms (R^2^ = 0.091); (c) Direct effect of sexual abuse and the presence of premenstrual symptoms. Numbers are displayed as regression coefficients (standard error). * *p* < 0.05; ***p < 0.001

The mediation analysis was adjusted over the following variables: age, education, financial burden and marital status. The results of the mediation analysis showed that suicidal ideation mediated the association between all types of child abuse and the presence of PMS (Table 3). Higher psychological (Beta = 0.21; *p* < 0.001), neglect (Beta = 0.02; *p* = 0.017), physical (Beta = 0.19; *p* < 0.001) and sexual (Beta = 0.20, *p* < 0.001) child abuse were significantly associated with higher suicidal ideation, which was significantly associated with the presence of PMS (Beta = 0.38, *p* = 0.001; Beta = 0.57, *p* < 0.001; Beta = 0.45, *p* < 0.001; and Beta = 0.50, *p* < 0.001) respectively. Finally, higher psychological (Beta = 0.17, *p* < 0.001), physical (Beta = 0.11, *p* = 0.024), but not sexual (Beta = 0.07, *p* = 0.198) child abuse was directly and significantly associated with the presence of PMS, whereas higher neglect (Beta = -0.06, *p* = 0.007) was significantly associated lower odds of having PMS (Figs. 1, 2, 3 and 4).

**Table 3:** *Mediation analyses results, taking each child abuse subscale as the independent variable, suicidal ideation as the mediator and the presence/absence of premenstrual symptoms as the dependent variable*

	Direct effect			Indirect effect		
	Beta	SE	p	Beta	Boot SE	Boot CI
Psychological abuse	0.17	0.05	< 0.001	0.08	0.03	0.03; 0.15*
Neglect	-0.06	0.02	0.007	0.01	0.01	0.004; 0.03*
Physical abuse	0.11	0.05	0.024	0.09	0.03	0.04; 0.16*
Sexual abuse	0.07	0.05	0.198	0.10	0.03	0.05; 0.18*

*indicates significant mediation. Direct effect refers to the direct association between child abuse and the presence of premenstrual symptoms without the effect of the mediator, whereas the indirect effect refers to the same association through the mediator (suicidal ideation).

## Discussion

Our study aimed to assess the mediating effect of suicidal ideation in the association between child abuse and PMS. The results reveal intricate relationships between these variables that resonate with existing literature on this topic. Child abuse, a known precursor to various mental health challenges, has been extensively documented in relation to its lasting impact [11, 14]. Specifically, we found strong associations between child psychological abuse and physical abuse, and PMS, consistent with prior research [4, 8, 42, 43].

Childhood trauma plays a significant role in the exacerbation of premenstrual symptoms. It is established that chronic stress and trauma linked to abuse can disrupt hormonal balance. This disruption might be magnifying the hormonal fluctuations responsible for PMS symptoms [24]. Moreover, individuals who have survived abusive experiences, often become more attuned to both emotional and physical changes, leading to heightened recognition and reporting of symptoms [25]. Additionally, coping strategies developed in response to abuse may indirectly contribute to hormonal imbalances and mood swings associated with PMS [26, 27].

Our study went beyond these established connections to reveal a novel insight: a history of abuse correlates with an increased likelihood of experiencing PMS symptoms, and this relationship is partially explained by the presence of suicidal ideation. Subsequently, suicidal ideation was associated with a higher risk of premenstrual symptoms, in alignment with previous data [15, 16, 18–20], emphasizing the significant role it plays in the complex interplay between child abuse and PMS. This mediation mechanism suggests that suicidal ideation resulting from early-life trauma may contribute to the exacerbation of premenstrual symptoms. This corresponds with theories linking psychopathology, childhood maltreatment, and subsequent mental health outcomes [21, 31]. Moreover, our study extended the existing literature by shedding light on the distressing impacts of child sexual abuse [11, 14]. Suicidal ideation emerged as a mediator in the relationship between sexual abuse and PMS, similar to findings, where emotional regulation played a role in the association between sexual abuse and PMS [12]. Additionally, our investigation revealed intriguing mediation patterns concerning neglect. Suicidal ideation acted as a mediator in the link between child neglect and PMS, akin to findings in literature where temperaments mediated this association [13]. These mediation mechanisms highlight the complex psychological responses that emerge in the context of child abuse and its impact on PMS.

Finally, this is our endeavor to explain the unexpected lack of a strong association between neglect and premenstrual symptoms, which may be attributed to several factors. Firstly, neglect may have a differential impact on stress levels when compared to more overt forms of abuse [44]. Neglect often involves emotional deprivation and a lack of caregiving, which, while distressing, may not consistently result in the chronic stress and trauma associated with PMS exacerbation. Secondly, the psychological resilience of individuals who have experienced neglect should not be underestimated. Some individuals may develop coping mechanisms and emotional strength that enable them to adapt to challenging circumstances [45], potentially mitigating the psychological and physiological effects of stress linked of PMS. When exploring coping mechanisms and protective factors in individuals who have experience neglect, it would be valuable for future studies to investigate specific strategies such as social support networks, self-regulation skills, and cognitive resilience. Understanding how these factors contribute to psychological resilience can shed light on potential avenues for interventions and support for individuals facing similar challenges. Thirdly, individual variability plays a substantial role; responses to neglect can vary greatly based on factors like genetics, and personal temperament [45, 46]. Additionally, reporting bias could come into play, as individuals may have difficulty recognizing or acknowledging neglect experiences, especially if they occurred during childhood [47], potentially leading to underreporting of childhood neglect in this study and other that tackled the association between childhood neglect and PMS. These complexities underscore the multifaceted nature of PMS and the need for further research to comprehensively study the relationship between neglect and this condition.

### Clinical implications

These findings carry valuable clinical implications. Recognizing the intricate relationships between child abuse, suicidal ideation, and PMS can aid healthcare providers in comprehensively addressing young women’s mental and reproductive well-being. Trauma-informed care, tailored interventions and awareness of potential connections between childhood maltreatment are essential in managing these complex challenges.

### Strengths and limitations

The study’s credibility is fortified by several key strengths that amplify its contributions. The inclusion of a robust sample of 915 participants enhances the statistical power and reliability of our findings. Supported by validated assessment tools, our study maintains accuracy and consistency in data collection.

Despite its strengths, the study is not without limitations. Firstly, its cross-sectional design limits our ability to establish causality or temporal relationships between the variables studied. The presence of confounding variables may influence the observed associations. Additionally, the reliance on self-report measures for variables like child abuse, premenstrual symptoms, and suicidal ideation introduces the possibility of recall bias and subjectivity. Furthermore, the use of retrospective reporting raises concerns about the reliability of participants’ recall of past experiences. Moreover, it is important to acknowledge limitations in the study design itself. The employment of snowball technique and the use of social media for participant recruitment may have unwittingly introduced biases. This method might not fully represent the diversity of the broader population, potentially impacting the external validity of our study’s findings. These methodological limitations should be considered when interpreting the results, particularly regarding the study’s generalizability and its applicability to populations beyond those accessed through snowball sampling or social media recruitment strategies.

### Recommendations

Healthcare providers should adopt trauma-informed care strategies, prioritizing the screening of signs indicating childhood maltreatment when addressing women’s mental and reproductive well-being. This proactive approach ensures a more comprehensive understanding of potential underlying trauma-related issues impacting conditions like PMS and suicidal ideation. Simultaneously, a collaborative effort among researchers and practitioners from diverse backgrounds is encouraged to craft tailored, multidisciplinary, and holistic approaches. Moreover, fostering education and awareness initiatives is crucial. Increased awareness among healthcare professionals, educators, and the wider public about nuanced connections between childhood abuse, mental health, and reproductive well-being can facilitate early identification, support, and preventative strategies. Implementing these measures not only enhances the effectiveness, empathy, and comprehensiveness of healthcare but also aims to improve the overall well-being of women affected by childhood trauma and its repercussions on mental health and reproductive experiences.

## Conclusion

Our study contributes to the growing understanding of the complex connections between child abuse, suicidal ideation, and premenstrual symptoms in 915 female adults, revealing significant mediation by suicide ideation. Psychological and physical abuse strongly correlate with increased premenstrual symptom severity, whereas neglect’s impact appears nuanced, prompting the need for further exploration. Our findings carry important clinical implications, emphasizing the need for trauma-informed care in healthcare. Implementing such approaches involves training healthcare professionals to recognize signs of childhood maltreatment, fostering safe and supportive environments, and tailoring interventions that acknowledge the intricate links between mental health and reproductive well-being. Moreover, targeted interventions focusing on coping mechanisms and emotional support for individuals who have experienced childhood abuse can significantly contribute to mitigating the impact of these traumatic experiences on premenstrual symptoms. This understanding lays the ground for future investigations, offering insights into effective intervention strategies, policy development, and a deeper understanding of the complex interplay between childhood maltreatment, premenstrual disturbances, and mental health outcomes.

## Data Availability

The datasets generated and/or analyzed during the current study are not publicly available due to the restrictions from the ethics committee but are available from the corresponding author on a reasonable request.
